# SMBE Secretary's Report

**DOI:** 10.1093/molbev/msae227

**Published:** 2024-11-01

**Authors:** Emmanuelle Lerat

**Affiliations:** Universite Claude Bernard Lyon 1, LBBE, UMR 5558, CNRS, VAS, Villeurbanne 69622, France

**Keywords:** SMBE, secretary, report

## Council Meetings

### Call to Order

President Stephen Wright called the council meeting to order at 8:45 AM Central Standard Time (CST) on July 7, 2024. In the presence of President Stephen Wright, Past-President Kateryna Makova, President-Elect Ziheng Yang, Councilors Maria C. Ávila Arcos, Christian Landry, Jeffrey Ross-Ibarra, Rebecca Zufall, Secretary Emmanuelle Lerat, Treasurer Aline Muyle, MBE Editors-in-Chief Brandon Gaut (ex officio) and Claudia Russo (ex officio), and GBE Editors-in-Chief Laura A. Katz (ex officio) and Adam Eyre-Walker (ex officio). In virtual attendance were Councilors Elena Gomez-Diaz and Juliana Vianna.

Non-council members in attendance: Chris Lapine (Allen Press) and Andy Seagram (Publisher OUP, MBE, and GBE).

Non-council members in virtual attendance: Lulu Stader (Executive Administrator).

### Treasurer's Report

Treasurer Aline Muyle presented the annual Treasurer's report, with details on journal and meeting revenue and expenses, with a projection on the year ahead. The society is globally financially healthy but the net asset is decreasing since 2022 and the projection for 2024 indicates that it is still going down showing that savings will be completely gone in six years. Council discussed several priorities moving forward to best serve our membership. Different ideas were proposed to increase the incomes, especially the donation, and to decrease the expenses with rethinking the organization of the annual meetings which is the biggest expense point. Full details can be found in the complete Treasurer's report.

### Secretary's Report

Secretary Emmanuelle Lerat briefly reviewed the Council's actions over the preceding year. Council voted on six motions between January 1 and July 7, 2024. Council approved: (i) the new OUP contract, (ii) the appointment of new AEs for MBE, (iii) the new formulation regarding APC waiver requests, (iv) the 2025 Article Processing Charges (APC), (v) the social Media & Highlights plan for SMBE journals for 2024 to 2027, and (vi) the commissioning of a search committee to replace Adam Eyre-Walker as EIC for GBE. Full details can be found in the complete Secretary report.

### Election of SMBE Officers

A nomination committee to propose candidates for the positions of President-elect and of two councilors was chaired by Laurent Duret with Soojin Yi, Mehmet Somel, Matt Webster, Ellen Leffler, and Yoko Satta as members, and Emmanuelle Lerat as ex officio (SMBE Secretary, as per by-laws). The membership of the society was informed by email of the committee composition in early 2024 and asked to nominate candidates for the positions. Council does and will continue to remind the nominating committee every year to prioritize gender and geographic representation among the candidates, but it maintains flexibility in how this is achieved by the committee.

The final selected nominees were Juliette de Meaux and Dmitri Petrov for President-elect, and Jesus Lozano-Fernandez, Aya Takahashi, Melissa Wilson, and Qi Zhou for Councilors. The membership of the society was issued online ballot details through Allen Press. In total, 493 members voted in this election out of 2,399 members invited. This corresponds to a 20.55% participation rate, which is higher than the previous year. The results of the election are that President-elect is Juliette de Meaux, and Councilors are Aya Takahashi and Melissa Wilson. The full nomination report is available as a separate document.

### Editors’/OUP/AP Reports

The Editors-in-Chief of MBE and GBE gave reports on the current states of their respective journals (see separate editors’ reports for full details). Different joint ventures for both journals happened during the past year (40th anniversary with logo contest, Why publish? initiative, improved transfer from MBE to GBE, pilot OUP transfer desk). For GBE, the submission numbers are still slightly down since pandemics and due to the competitive market. However, the IF remains stable and the rankings for “Evolutionary Biology” (16 out of 54) and “Genetics” (20 out of 52) have improved. There is a need to work to maintain the number of submission and increase the number of citation while maintaining commitment to inclusive publishing. MBE continues to be in a very strong position with an IF at 11 in 2023 making MBE ranking fourth among 54 Evolutionary Biology journals.

### Other Activities

#### Future Annual Meetings

SMBE 2025 will be in Beijing, China. SMBE 2026 will be in Copenhagen, Denmark. It was decided that the two meetings should be hybrid to allow better accessibility.

#### IDEA Task Force, Proposals

The call for IDEA proposals resulted in the funding of one proposal: Resources for Inclusive Education (RIE2) (proposed by Yaamini Venkataraman), in addition to lab collaboration with students funded to come to SMBE 2024. An outreach symposium was organized at SMBE 2024. Currently, the task force is working toward completing a guide for inclusive conferences to ensure that all SMBE meetings are organized following IDEA.

#### Career Awards

Thirty-two people were supported by this award to attend the SMBE 2024 meeting in Puerto Vallarta, Mexico.

#### Early Career Award

This award is given to outstanding members of the SMBE community who are in the early stages of an independent research career. The 2024 recipient was Parul Johri from the University of North Carolina (Chapel Hill, USA).

#### Mid-Career Award

This award is intended for outstanding members of the SMBE community who are in the midst of their research careers. The 2024 recipient was Deepa Agashe from the National Center for Biological Sciences (Bangalore, India).

#### Lifetime Achievement Award

This award is intended for outstanding senior members of the SMBE community. The 2024 recipient was Marcus Feldman from Stanford University (USA).

#### Graduate Student Excellence Awards

This symposium provides a forum for young investigators to showcase their exemplary research and continues to be a highlight of the SMBE annual meeting. We celebrate all of the candidates in this symposium. The awardees were:

Christopher Blake (Monash University, Australia)
Sung-Ya Lin (University of Pennsylvania, USA)
Jaison Jeevan Sequeira (Mangalore University, India)
Maria Jose Palma Martinez (Center for Genomic Sciences, UNMAM, Mexico)
Ben Michael Moran (Stanford University, USA)
Meaghan Marohn (UC Berkley, USA)
Mariela Tenorio (Center for Genomic Sciences, UNMAM, Mexico)
Siliang Song (University of Michigan, USA).

#### Best Graduate Student Paper Award

These awards provide recognition for outstanding student papers in both SMBE journals. For MBE, the committee selected one winner.

Winner: Xing Long: “Independent Evolution of Sex Chromosomes and Male Pregnancy-Related Genes in Two Seahorse Species”.

For GBE, the committee selected one winner.

Winner: Yawako Kawaguchi “Extensive Copy Number Variation Explains Genome Size Variation in the Unicellular Zygnematophycean Alga, *Closterium peracerosum-strigosum*-littorale Complex”.

#### SMBE Undergraduate Mentorship Awards 2024

These awards were granted to ten undergraduate students to attend the SMBE 2024 meeting in Puerto Vallarta, Mexico. In this program, each student is paired with a mentor to assist them throughout the SMBE meeting.

## Business Meeting

This year's business meeting took place on July 9, 2024, during the SMBE meeting in Puerto Vallarta, Mexico. The President recognized the efforts of the local organizing committee for the meeting, emphasizing the significance of the first SMBE conference held in mainland Latin America. He provided a financial overview of the society, detailing the various awards presented during the event. Additionally, the President offered insights into membership categories and updates, as well as the benefits of being a member. He shared statistics regarding our journals, including impact factors and submission rates. The election results were announced, along with the locations for SMBE 2025 and 2026. The President also outlined the upcoming regional and satellite meetings, and highlighted the work of the IDEA task force.

## Data Availability

No data used.

